# Delayed phases of contrast MRI, can it be valuable in multiple sclerosis active phase diagnosis?

**DOI:** 10.22088/cjim.11.4.432

**Published:** 2020

**Authors:** Hossein Pajouhan-Far, Naser Qaemian, Karimollah Hajian-Tilaki, Mehrdad Nabahati, Payam Saadat, Raheleh Mehraein

**Affiliations:** 1 Department of Radiology, Babol University of Medical Sciences, Babol, Iran; 2 Social Determinants of Health Research Center, Health Research Institute, Babol University of Medical Sciences, Babol, Iran; 3 Mobility Impairment Research Center, Health Research Institute, Babol University of Medical Sciences, Babol, Iran

**Keywords:** Multiple sclerosis, Plaque enhancement, Plaque size, Plaque count, Contrast M

## Abstract

**Background:**

Observing the enhancing plaques in magnetic resonance imaging (MRI) is one of the most valuable diagnostic modalities in confirming the diagnosis of multiple sclerosis (MS), its recurrence and for better detection of active disease. Since active lesions discovery can improve designating diffusion in time diagnosis of MS and controlling disease activity, and there is not any definite time for delay image acquisition, therefore, the aim of the current study was to evaluate the enhancement of MS plaques in different delayed phases.

**Methods:**

In this interventional study, after receiving written consent, 40 MS patients with at least one enhancing plaque in a previous MRI were evaluated in Babol Ayatollah Rouhani Hospital. Gadolinium was injected to all patients at the dose of 0.1 mg/kg, and MRI was taken at 5 and 15 minutes. The results were analyzed using SPSS 23. A p<0.05 was considered as significant level.

**Results:**

The mean of plaque signal intensity was 1190.20 and 1349.60 at 5 and 15 min, respectively, and this difference was significant (p<0.001). Moreover, the mean of plaque total size was 5.16 cm and 7.04 cm at 5 and 15 min with significant difference, respectively (p<0.001). The mean of plaque number was 1.92 and 2.58 at 5 and 15 min, respectively, which was significantly different (P<0.001).

**Conclusion:**

The results indicated improvement in detection of MS plaques in images taken in the delayed phase compared to those in the early phase. The plaque intensity, size and number were significantly higher in the delayed phase (15 min), than early phase (5 min).

Multiple sclerosis (MS) as the most common inflammatory demyelinating diseases (IDDs) of the central nervous system is one of the most common causes of disability in young adults after trauma (1, 2). It is three times more common in women than in men (3, 4). The disease is initially diagnosed clinically and its definitive diagnosis was made by confirming the diffusion of central nervous system lesions based on clinical evidence alone, or in combination with magnetic resonance imaging (MRI) evidence on the basis of the time and location of the lesion. History and careful clinical examination are the most important part of diagnosis, and MRI is used as a screening test to confirm clinical findings (5). Brain or spinal plaques are found in MRI imaging findings of MS. Pathologically, these plaques are composed of a demyelinated area with relative axonal preservation. Some studies have indicated that the axonal injury is a prominent pathological feature of MS, this axonal loss can occur in the demineralization process or separate process (6, 7). The McDonald's diagnostic criteria are specifically used based on the time and location of the lesion to approve the evidence by MRI. 

However, the primary purpose of this diagnostic criterion is not to distinguish MS from other neurological disorders (8).

Two criteria can be used to diagnose MS lesions: 1) Based on location, the presence of one or more lesions in at least 2 MS-typical regions such as periventricular, juxtacorticular, infratentorial or spinal cord or lesion extending to other CNS areas through reattack and 2) Based on time, asymptomatic lesions on the MRI with or without gadolinium enhancement at any time, or new lesions with gadolinium enhancement on the follow-up T2 MRI regardless of the initial scan time or with a new clinical attack. It should be noted that the McDonald's criteria can only be used if the patient has been carefully evaluated clinically. The delayed phase in plaque enhancement is performed during 15-60 min. For this reason, no specific gold standard time is mentioned (9). The aim of this study was to evaluate the diagnostic accuracy of MS based on MRI findings with intravenous contrast and to better understand the changes in its delayed phase.

## Methods

This interventional study was performed on 40 MS patients based on clinical evidence and previous MRI findings. Inclusion criterion was confirmation of the presence of MS based on MRI examinations and findings with at least one enhancing plaque. Patients with MRI contraindications such as having a metal prosthesis, fear of MRI, lack of enhancing plaque and no consent were excluded from the current study. Demographic data including age, gender, residence status, education status of patients were obtained. MRI imaging was performed using General Electric, Signa, 1.5 Tesla system. 

The MRI was performed with gadolinium diethylenetriaminepentaacetic acid (Gd-DTPA) intravenous contrast in all patients. The results of contrast MRI including the size and number of plaques in the early (5min) and delayed (15min) phases were recorded in the checklist of the study. Contrast injection was conducted according to previous studies, and 0.1 mmol/kg Gd-DTPA was administered by intravenous bolus injection (10). Early and delayed sequences were prepared at 5 and 15 min after contrast injection, respectively.

**Statistical analysis:** Data were analyzed by SPSS 23 using chi-square and t-test. Correlation between the sequences was evaluated by Pearson correlation analysis. A p<0.05 was considered as significant level.

## Results

30.65±8.82 (15-48) years were evaluated. Of these, 27 cases were <35 years and 29 were females. The information on patients' age and gender is illustrated in table 1.

**Table 1:** The demographic characteristics of the patients

**Variables**	
Age (Year) mean±SD >35 N(%) ≥35 N(%)	30.65±8.82 27(67.5) 13(32.5)
Sex (%) Male Female	11(27.5) 29(72.5)
Level of Education Master’s degree and higher Bachelor Associate degree Diploma and lower	1 12 3 24
Duration of disease (mean±SD)	6±5.1
Comorbid Disease	1
Area (%)N Urban Rural	22(55) 18(45)

The mean of plaque signal intensity, total size and number at 5 and 15 min are shown in table 2, indicating a significant difference between these indices at 5 and 15 min.

**Table 2:** Comparison of plaque characteristics by time of MRI in patients

**Variable**	**Time of MRI** **SD** **±** **Mean**		**Pvalue**
	**5 min**	**15 min**	
Plaque signal intensity	1191.20±145.28	1349.60±125.26	<0.001
Total size of plaque (cm)	5.16±2.57	7.04±2.91	<0.001
Number of plaque	1.92±0.76	2.58±1.01	<0.001

**Figure 1:**
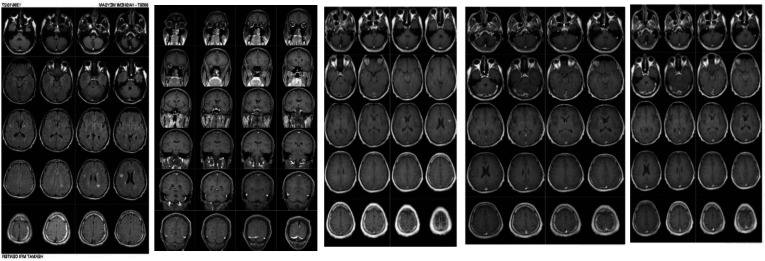
Brain MRI with and without contrast: a) Axial FLAIR images show several pericallosal and juxtacortical plaques of MS as hyperintense lesions. b, c, d) Sagittal and axial T1 postgadolinium images at 5 ,7 and 8 minutes after injection display mild plaque enhancement at right internal capsule, left parietal and left high frontal juxtacortical white matter (WM), respectively. e) Axial delayed post contrast image exhibits significant enhancement at mentioned plaques and a new lesion in the right thalamus

Figure 1 illustrates the brain MRI with and without contrast. In this figure, plaques are represented before and after injection and plaque enhancement. The mean signal intensity, size and number of plaques at 5th and 15th minutes were evaluated based on the age and gender. At 5th min of MRI, females had significantly higher plaque signal intensity than males. In addition, the total plaque size was significantly higher in patients <35 than ≥35 years at 15th min of MRI. The number of plaques was also significantly higher in persons <35 than ≥35 years at 15th min of MRI (table 3).

**Table 3:** Evaluation of plaque signal intensity at 5 and 15 min by gender and age of patients

**Variables**		**Plaque signal intensity** **SD±Mean**		**P-value**
		**5 Min**	**15 Min**	
Sex	Male	1100.73±185.83^a^	1293.36±145.07	<0.001
	female	1225.52±112.38^b^	1370.93±112.36	<0.001
Age	<35	1185.81±121.02	1360.15±114.30	<0.001
	≥35	1202.38±191.48	1327.69±148.05	<0.001
		Total size of plaque (cm) SD±Mean		P-value
		5 min	15 min	
Sex	Male	5.41±1.97	7.16±2.27	<0.001
	Female	5.06±2.79	7.00±3.16	<0.001
Age	<35	5.64±2.73	7.71±2.85^a^	<0.001
	≥35	4.15±1.92	5.65±2.63^b^	<0.001
		Number of plaque Mean±SD		P-value
		5 Min	15 Min	
Sex	Male	2.00±0.63	2.73±1.01	<0.001
	female	1.90±0.82	2.52±1.02	<0.001
Age	<35	2.07±0.83	2.81±1.04^a^	<0.001
	≥35	1.62±0.51	2.08±0.76^b^	<0.001

a and b, are significant

## Discussion

According to the findings of the current study, in the delayed phase of contrast-enhanced MRI (15 min), the number of plaques observed, total plaque size and signal intensity of the plaques were significantly higher than those in the early phase of contrast-enhanced MRI (5 min). Uysal et al. in 2007, found that the plaque enhancement was significantly higher in delayed images at 5 and 10 min than in images taken at 0 min (11). They prepared MRI images after the injection of 0.5 and 1 mmol/L of gadolinium at 0, 5 and 10 min while in our study, the images were taken at early time of 5 min and delayed time of 15 min. Moreover, the 1mmol/L gadolinium contrast was used in the ongoing study.

These items were different between their and our studies; however, in general, their results are in line with ours. In some studies, the diagnostic value of delayed-phase MRI in MS patients has also been investigated (12, 13). The dose of gadolinium used in these studies was similar to that in the present study whereas the delayed phase was after one hour in the study of Bagheri et al. and was after 15 min in the current study. The early phase was 5 min in both studies. The results of Bagheri et al. suggested an increased sensitivity to detect plaques one hour after contrast injection.

In the ongoing study, the enhancement and detection of plaques in the delayed phase of 15 min were significantly higher than in the early phase of 5 min. In terms of the delayed phase, their study was significantly different from ours, but the results of both studies were similar and consistent. In the studies of Silver et al., the sensitivity of the short-phase (20-40 min) and long-phase (40-60 min) time after gadolinium injection was evaluated in MS patients (14, 15). There were no significant differences in plaque detection between two phases in their studies. Our study was different from their studies in determining the duration of MRI after gadolinium injection as well as their results were inconsistent with ours. In addition, in terms of gender, in the early phase of contrast-enhanced MRI (5 min), females showed significantly greater signal intensity than males. This finding was not significant for plaque size and number as well as for the delayed phase. 

In a study by Hashemi et al., the MRI was performed at 30 s as well as 10, 5 and 15 min after gadobutrol injection, and the signal intensity, size and enhancement were measured for each plaque. The maximum number of enhanced plaques was at 15 min. The size and signal intensity of the lesion also significantly increased at this time (16), which is similar to the present study. In the current study, phase zero was not investigated in contrast MRI, and since T1 and T2 images had no contrast, phase 5 was considered as phase zero to compare the enhancement and number of plaques. On the other hand, to minimize the impact of the patient's transposition errors for plaque comparison, the intravenous contrast material was administered to patients before lying in the bed prior to MRI onset. However, different doses of gadolinium were not considered, the delayed phase was limited to 15 min after contrast injection and the delayed phases of 30 min and 1 hour were not evaluated to prevent prolonged study time and to avoid patients’ transposition during MRI. The results of the present study showed improvement in the detection of MS plaques in the images prepared in the delayed phase of 15 min after injection of 0.1 mmol/kg gadolinium compared to those in the early phase of 5 min.

The plaque enhancement, size and number were significantly higher in the delayed phase (15 min) than in the early phase (5 min). It is therefore better to use delayed-phase images, 15 minutes after injection of 0.1 mmol/kg gadolinium, in the detection of plaque size in MS patients who previously had at least one enhancing plaque.
